# Supplementary material for: Conserved RNA-binding specificity of polycomb repressive complex 2 is achieved by dispersed amino acid patches in EZH2
Source: eLife. 2017 Nov 29;6:e31558. doi: 10.7554/eLife.31558 (PMC5706960; doi:10.7554/eLife.31558)
Supplement: Supplementary file 1. [file elife-31558-supp1.docx]

**Supplemental File 1. List of all proteins used in this study**

| species | number of subunits | Name | Composition (e*ac*h peptide separated by ";") | Expression system | mentioned in Figure |
| --- | --- | --- | --- | --- | --- |
| *ct*PRC2 | 3 | 3m *ct*PRC2 wild type | *ct*EZH2-VEFS(*ct*SUZ12); *ct*EED | *S. cerevisiae* | Figure 1 |
|  | 3 | 3m *ct*PRC2 mutants | *ct*EZH2-VEFS(*ct*SUZ12); *ct*EED | *S. cerevisiae* | Figure 3 |
|  | 2 | regulatory moiety | *ct*EZH2 (N-half); *ct*EED | *E. coli* | Figure 2 |
|  | 1 | *ct*EED | *ct*EED | *E. coli* | Figure 2 |
|  | 2 | minimal RNA binding complex and its mutants | EBD-BAM(*ct*EZH2); *ct*EED | *E. coli* | Figure 2 and 3 |
|  | 1 | VEFS(SUZ12) | VEFS(*ct*SUZ12) | *E. coli* | Figure 2 |
|  | 2 | VEFS-SANT2L | VEFS(*ct*SUZ12); SANT2L(*ct*EZH2) | *E. coli* | Figure 2 |
|  | 1 | CXC-SET | CXC-SET(*ct*EZH2) | *E. coli* | Figure 2 |
| *hs*PRC2 | 5 | 5m *hs*PRC2 Wild type | *hs*EZH2; *hs*SUZ12; *hs*EED; *hs*RBBP4; *hs*AEBP2 | inse*ct* cell | Figure 4A, sup Fig. 5 |
|  | 4 | 4m *hs*PRC2 Wild type | *hs*EZH2; *hs*SUZ12; *hs*EED; *hs*RBBP4 | inse*ct* cell | sup Fig. 1 |
|  | 3 | 3m *hs*PRC2 Wild type | *hs*EZH2; *hs*EED (81-441); *hs*SUZ12-VEFS (545-726; S583D) | inse*ct* cell | Figure 4A and 5, sup Fig. 3 |
|  | 3 | 3m *hs*PRC2 mutants | *hs*EZH2; *hs*EED (81-441); *hs*SUZ12-VEFS (545-726; S583D) | inse*ct* cell | Figure 6 |
|  | 3 | 3m *hs*PRC2 wild type | *hs*EZH2-VEFS(*hs*SUZ12); *hs*EED | *S. cerevisiae* | Figure 4B, sup Fig. 2 |
|  | 3 | 3m *hs*PRC2 mutants | *hs*EZH2-VEFS(*hs*SUZ12); *hs*EED | *S. cerevisiae* | Figure 4B, sup Fig. 2 |
|  | 2 | SUZ12ΔVEFS-RBBP4 | *hs*SUZ12ΔVEFS; *hs*RBBP4 | *S. cerevisiae* | Figure 4B, sup Fig. 2 |
| *ac*/*hs*PRC2 | 3 | 3m *ac*/*hs*PRC2 wild type | *ac*EZH2; *hs*EED (81-441); *hs*SUZ12-VEFS (545-726; S583D) | inse*ct* cell | sup Fig. 4 |
|  | 3 | 3m *ac*/*hs*PRC2 mutants | *ac*EZH2; *hs*EED (81-441); *hs*SUZ12-VEFS (545-726; S583D) | inse*ct* cell | sup Fig. 4 |
| *mm*PRC2 | 4 | 4m *mm*PRC2 wild type | *mm*EZH2; *hs*SUZ12; *hs*EED; *hs*RBBP4 | inse*ct* cell | sup Fig. 1 |
|  | 4 | 4m *mm*PRC2 mutant | *hs*EZH2; *hs*SUZ12; *hs*EED; *hs*RBBP4 | inse*ct* cell | sup Fig. 1 |
