# Supplementary material for: Conserved RNA-binding specificity of polycomb repressive complex 2 is achieved by dispersed amino acid patches in EZH2
Source: eLife. 2017 Nov 29;6:e31558. doi: 10.7554/eLife.31558 (PMC5706960; doi:10.7554/eLife.31558)
Supplement: Supplementary file 2. [file elife-31558-supp2.docx]

**Supplementary File 2. Mutations in *ct*PRC2 that had < 3-fold effect on affinity for (G4A4)4 RNA**

| Subunit | Domain | Mutants  (residue numbering according to Jiao and Liu, 2015) |
| --- | --- | --- |
| EZH2 | CXC | [K649A K651A R658G R659S K660G K661S] |
|  |  | [R658G R659S K660G K661S K723A R744A] |
|  | SANT1L | [K367A R368A K417A] |
|  |  | [R460A] |
|  | SANT2L | [R623A W626A H629A R630A K631A] |
| SUZ12 and EED | VEFS(SUZ12) and EED | [SUZ12 (K2643A R2644A R2646A); EED (R13A K349A K351A K512A R515A)] |
| EED | EED | [R526A] |

Mutations shown in the bracket ([…]) were made in one mutant protein. Mutant proteins (all 3m *ct*PRC2) were expressed as two polypeptides (EZH2-VEFS and EED) in *S. cerevisiae* strain CB010 and purified using Ni-NTA agarose resin followed by resolving using Superdex 200 size-exclusion chromatography column. *K*_d_^app^ was determined by EMSA**.**
